# The STAGED-PKD 2-Stage Adaptive Study With a Patient Enrichment Strategy and Treatment Effect Modeling for Improved Study Design Efficiency in Patients With ADPKD

**DOI:** 10.1016/j.xkme.2022.100538

**Published:** 2022-08-27

**Authors:** Ronald D. Perrone, Ali Hariri, Pascal Minini, Curie Ahn, Arlene B. Chapman, Shigeo Horie, Bertrand Knebelmann, Michal Mrug, Albert C.M. Ong, York P.C. Pei, Vicente E. Torres, Vijay Modur, Ronald T. Gansevoort

**Affiliations:** 1 Division of Nephrology, Tufts Medical Center, Tufts University School of Medicine, Boston, Massachusetts; 2 Eloxx Pharmaceuticals, Watertown, Massachusetts; 3 Sanofi, Chilly Mazarin, France; 4 Department of Internal Medicine, Seoul National University, Seoul, Republic of Korea; 5 Department of Medicine, University of Chicago, Chicago, Illinois; 6 Department of Urology, Juntendo University Graduate School of Medicine, Tokyo, Japan; 7 Université de Paris, AP-HP, Service de Néphrologie, Hôpital Necker-Enfants Malades, Paris, France; 8 Department of Medicine, University of Alabama at Birmingham, Birmingham, Alabama and Department of Veterans Affairs Medical Center, Birmingham, Alabama; 9 Academic Nephrology Unit, Department of Infection Immunity & Cardiovascular Disease, University of Sheffield Medical School, Sheffield, United Kingdom; 10 Division of Nephrology, University of Toronto, Toronto, Ontario, Canada; 11 Division of Nephrology and Hypertension, Mayo Clinic, Rochester, Minnesota; 12 Department of Nephrology, University Medical Center Groningen, The Netherlands

**Keywords:** Autosomal dominant polycystic kidney disease, modeling, study design, total kidney volume, venglustat

## Abstract

**Rationale & Objective:**

Venglustat, a glucosylceramide synthase inhibitor, inhibits cyst growth and reduces kidney failure in mouse models of autosomal dominant polycystic kidney disease (ADPKD). STAGED-PKD aims to determine the safety and efficacy of venglustat and was designed using patient enrichment for progression to end-stage kidney disease and modeling from prior ADPKD trials.

**Study Design:**

STAGED-PKD is a 2-stage, international, double-blind, randomized, placebo-controlled trial in adults with ADPKD (Mayo Class 1C-1E) and estimated glomerular filtration rate (eGFR) 45-<90 mL/min/1.73 m^2^ at risk of rapidly progressive disease. Enrichment for rapidly progressing patients was identified based on retrospective analysis of total kidney volume (TKV) and eGFR slope from the combined Consortium for Radiologic Imaging Studies of Polycystic Kidney Disease and HALT Progression of Polycystic Kidney Disease A studies.

**Setting & Participants:**

Target enrollment in stages 1 and 2 was 240 and 320 patients, respectively.

**Interventions:**

Stage 1 randomizes patients 1:1:1 to venglustat 8 mg or 15 mg once daily or placebo. Stage 2 randomizes patients 1:1 to placebo or venglustat, with the preferred dose based on stage 1 safety data.

**Outcomes:**

Primary endpoints are TKV growth rate over 18 months in stage 1 and eGFR slope over 24 months in stage 2. Secondary endpoints include: annualized rate of change in eGFR from baseline to 18 months (stage 1); annualized rate of change in TKV based on magnetic resonance imaging from baseline to 18 months (stage 2); and safety, tolerability, pain, and fatigue (stages 1 and 2).

**Limitations:**

If stage 1 is unsuccessful, patients enrolled in the trial may develop drug-related adverse events that can have long-lasting effects.

**Conclusions:**

Modeling allows the design and powering of a 2-stage combined study to assess venglustat’s impact on TKV growth and eGFR slope. Stage 1 TKV assessment via a nested approach allows early evaluation of efficacy and increased efficiency of the trial design by reducing patient numbers and trial duration.

**Funding:**

This study was funded by Sanofi.

**Trial registration:**

STAGED-PKD has been registered at ClinicalTrials.gov with study number NCT03523728.

**Plain-Language Summary**

Patients with autosomal dominant polycystic kidney disease experience kidney enlargement due to growth of kidney cysts, which eventually leads to kidney failure. Venglustat, a new oral therapy, may prevent kidney cyst formation and preserve kidney function. This paper describes the design of a single, 2-stage study that evaluates the safety and efficacy of venglustat in patients with autosomal dominant polycystic kidney disease who have a high cyst burden and decreased kidney function. This study design enables the assessment of venglustat in one efficient, short-duration trial. It uses a strategy that enables the study population to be enriched with patients who have rapidly progressing disease. The number of patients included is based on modeling the relationship between 2 biomarkers of disease severity.

Autosomal dominant polycystic kidney disease (ADPKD) is a monogenic disease characterized by development of fluid-filled kidney cysts, kidney enlargement, hypertension, and eventual progression to kidney failure.^1^^,^^2^ Glycosphingolipids (GSLs) are lipid molecules with important roles as structural components of cellular membranes and cell-signaling regulators.^3^ GSLs are enriched in certain microdomains of kidney tubule cell membranes, including primary cilia, where pathogenic GSL accumulation can disrupt ciliary signaling, leading to cyst formation.^4^ GSLs accumulate in ADPKD cells via increased glucosylceramide synthase (GCS) activity and increased *de novo* ceramide synthesis (Fig 1).^4^

**Figure 1:**
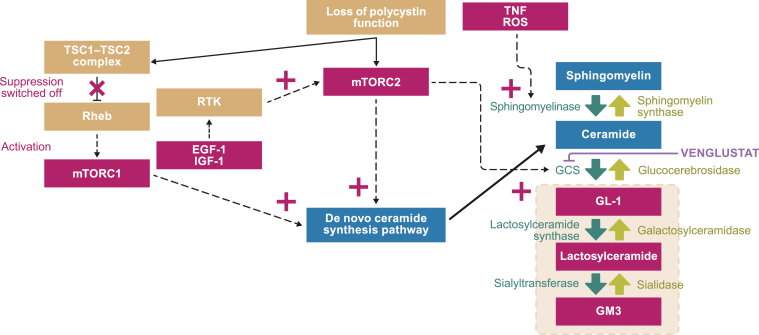
GSL biosynthesis in ADPKD and potential effect of the GCS inhibitor venglustat. Adapted from Natoli et al.^4^ Loss of polycystin function disrupts the TSC complex, and consequently suppression of Rheb is inactivated, leading to activation of mTORC1. mTORC1 activation increases de novo ceramide synthesis. Polycystin dysregulation also activates mTORC2, which also promotes de novo ceramide synthesis and increases GL-1 by increasing GCS production. Beyond polycystin disruption, other factors may impact GSL accumulation in ADPKD, including growth factor activation (eg, EGF 1 or IGF 1) of mTORC2 or cytokine- and ROS-mediated activation of sphingomyelinase activity. Red boxes show molecules that are upregulated in cystic kidneys compared with normal kidneys. Yellow box overlay indicates GSL accumulation that could be attenuated/stopped with a GCS inhibitor. Abbreviations: ADPKD, autosomal dominant polycystic kidney disease; EGF, epidermal growth factor; IGF, insulin-like growth factor; GCS, glucosylceramide synthase; GL-1, glucosylceramide; GM3, monosialodihexosylganglioside; GSL, glycosphingolipid; mTORC, mammalian target of rapamycin complex; Rheb, Ras homolog enriched in brain; ROS, reactive oxygen species; RTK, receptor tyrosine kinase; TNF, tumor necrosis factor; TSC, tuberous sclerosis.

Venglustat is a once-daily, oral investigational GCS inhibitor (Fig 1) that may reduce GSL production.^4^ In mouse models of ADPKD, lowering glucosylceramide levels by ≥70% with venglustat was associated with reduced cyst growth, increased cyst cell differentiation, and preservation of kidney function.^4^ In a phase 1 study of healthy individuals, venglustat reduced plasma glucosylceramide, which resulted in venglustat receiving orphan drug designation for ADPKD from the US Food and Drug Administration and the European Medicines Agency.^5^, ^6^, ^7^

Designing interventional trials in ADPKD is complex. A patient’s current disease state (ie, kidney function) and risk for progression (ie, cyst burden) should be the principal considerations for inclusion criteria in ADPKD interventional trials. Patients should be enrolled on the basis that treatment is anticipated to be effective; however, the study population should also be enriched for individuals at greater risk of disease progression and will reach study endpoints during the timeframe of the trial.^8^

The trajectory of kidney function decline is strongly associated with Mayo Imaging Class 1 (uniform distribution of cysts throughout the kidneys), which categorizes patients in 1 of 5 groups based on their height-adjusted total kidney volume (TKV) at a given age.^9^^,^^10^ TKV can be measured reliably and is a recognized prognostic enrichment biomarker in patients with ADPKD, increasing over time to reflect kidney cyst enlargement, preceding loss of kidney function, and identifying patients at higher risk of developing kidney failure.^9^^,^^11^, ^12^, ^13^ TKV growth rate is accepted as a reasonably likely surrogate endpoint for ADPKD progression trials by the Food and Drug Administration and may provide evidence for accelerated drug approval, though subsequent determination of the intervention’s effect on a clinical endpoint, such as eGFR, is necessary if an effect on the reasonably likely surrogate endpoint is demonstrated.^8^^,^^14^^,^^15^

The STAGED-PKD trial (**S**tudy **T**o **A**ssess **G**lucosylceramide synthase inhibitor **E**fficacy in A**DPKD**; NCT03523728) is an international, multicenter, randomized, double-blind, placebo-controlled study designed to characterize the efficacy, safety, tolerability, and pharmacokinetics of venglustat in patients with rapidly progressing ADPKD. This paper describes the adaptive 2-stage design of STAGED-PKD that used (1) a patient enrichment strategy that identified a rapid progressor population for inclusion in the trial and (2) first-time use of modeling exploring the relationship between TKV and eGFR. STAGED-PKD tests the hypothesis that GCS inhibition by venglustat, at a dose with a favorable safety and tolerability profile, may be a viable treatment to slow cyst growth and preserve kidney function in patients with rapidly progressing ADPKD. This enables the assessment of venglustat on TKV and eGFR in one efficient, short-duration trial.

## Methods

### Enrichment of Patients at Risk of Rapidly Progressing ADPKD

Prognostic enrichment was used for the primary analysis population to select patients at risk for rapidly progressing ADPKD based on age (18-50 years), chronic kidney disease stage (G2–3A), and Mayo Imaging Classification (1C–1E). Using prognostic enrichment to define eligibility criteria enables the detection of treatment benefit on TKV and eGFR in the same population, avoiding the need to perform separate trials in patients with early- and late-stage ADPKD.

To enrich for patients with rapidly progressing ADPKD, a retrospective analysis was performed of data from 2 published prospective, longitudinal studies, the Consortium for Radiologic Imaging Studies of Polycystic Kidney Disease (CRISP; NCT01039987) study and HALT Progression of Polycystic Kidney Disease Study A (HALT-PKD A; NCT00283686; Item S1).^16^, ^17^, ^18^ Patients included in CRISP and HALT-PKD A were used (Table S1) because databases from both were available in the National Institute of Diabetes and Digestive and Kidney Diseases central repositories, and both studies included patient populations similar to the STAGED-PKD target population. These datasets were obtained after receiving ethics committee approval. Patients from CRISP and HALT-PKD A were stratified by Mayo Imaging Class and, within each class, TKV growth rate was plotted against eGFR rate of decline.

### Modeling the Relationship Between TKV Growth and eGFR Decline

Although TKV growth rate is accepted as a reasonably likely surrogate endpoint for ADPKD progression trials by the Food and Drug Administration, there is currently no fixed definition of what constitutes a substantial treatment effect on TKV. Therefore, existing data were used to develop a quantitative understanding of the relationship between TKV and kidney function outcomes. Assuming that a 30% treatment effect on the rate of eGFR decline would be verifiable in a postmarketing confirmatory trial (based on the outcomes of the tolvaptan TEMPO 3:4 and REPRISE studies), an effect on TKV growth that could be reasonably translated into a 30% improvement in eGFR decline was targeted as constituting a substantial treatment effect in subsequent modeling.^19^^,^^20^

**Figure 2:**
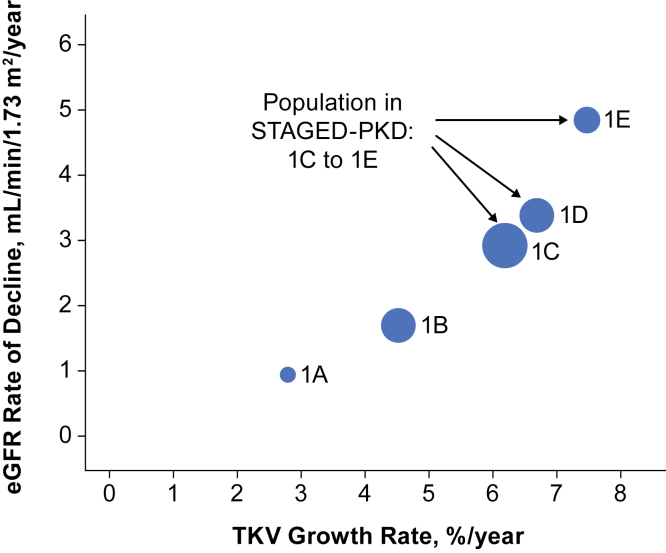
Class-level data from the CRISP and HALT-PKD A studies. Based on retrospective analysis of 2 historical studies. For each class, mean TKV growth rate is plotted against mean eGFR rate of decline. The size of each bubble is proportional to the sample size. Abbreviations: CRISP, Consortium for Radiologic Imaging Studies of Polycystic Kidney Disease; eGFR, estimated glomerular filtration rate; HALT-PKD, HALT Progression of Polycystic Kidney Disease; STAGED-PKD, study to assess glucosylceramide synthase inhibitor efficacy in ADPKD; TKV, total kidney volume.

All patients who were included in CRISP (N = 241) or HALT-PKD A (N = 558) were considered. Seventy-seven patients were excluded because of a lack of baseline or postbaseline TKV or eGFR data; therefore, 722 patients were included in this retrospective analysis (Item S2, Fig S1). TKV growth rate increased with increasing Mayo Class during study follow-up (Table 1, Fig 2). Similarly, eGFR declined with increasing Mayo Class during follow-up (Table 1, Fig 2).

**Table 1:** TKV Growth Rate and eGFR Rate of Decline in CRISP and HALT-PKD A Studies, by Mayo Class

Parameter	Mayo Class					*P*
	1A (N = 44)	1B (N = 165)	1C (N = 251)	1D (N = 167)	1E (N = 95)	
TKV growth rate, %/y						
Estimate	2.8	4.5	6.2	6.7	7.5	< 0.001
95% CI	2.0-3.5	4.0-5.0	5.7-6.7	6.1-7.3	6.5-8.5	
eGFR rate of decline, mL/min/1.73 m^2^/y						
Estimate	0.96	1.71	2.96	3.40	4.87	< 0.001
95% CI	0.30-1.61	1.41-2.02	2.68-3.23	3.04-3.76	4.24-5.50	

*Note*: Mean TKV growth rate was estimated from a linear mixed-effect model on log_10_(TKV). Mean eGFR rate of decline estimated from a linear mixed-effect model.

Abbreviations: CI, confidence interval; CRISP, Consortium for Radiologic Imaging Studies of Polycystic Kidney Disease; eGFR, estimated glomerular filtration rate; HALT-PKD, HALT Progression of Polycystic Kidney Disease; TKV, total kidney volume.

### Retrospective Analysis of Individual TKV and eGFR Data From CRISP and HALT-PKD A

**Figure 3:**
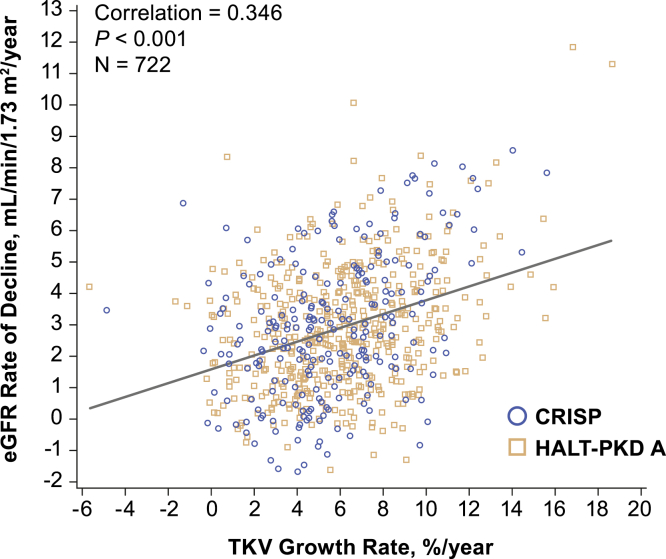
Correlation between TKV growth and eGFR decline based on individual-level data from CRISP and HALT-PKD A. Predicted eGFR rate of decline based on a model predicting eGFR at time t as a function of TKV growth rate and adjusted for baseline eGFR and age. Abbreviations: CRISP, Consortium for Radiologic Imaging Studies of Polycystic Kidney Disease; eGFR, estimated glomerular filtration rate; HALT-PKD, HALT Progression of Polycystic Kidney Disease; TKV, total kidney volume.

The Pearson correlation between individual rates of TKV growth and eGFR decline showed a significant correlation (*P* < 0.001; Fig 3), although the correlation coefficient (0.35) was weak. A statistical model predicting future eGFR at time t as a function of baseline eGFR, age, and TKV growth rate was also built (Item S3, Table S2**)**. A significant interaction between the TKV growth rate and time (*P* < 0.001) indicated that the rate of eGFR decline significantly increased with increasing TKV growth rate.

Based on this model, the predicted rate of decline in eGFR associated with different TKV growth rates was calculated (Table 2). This suggested that a 50% reduction in TKV growth rate would be associated with a 20% to 30% reduction in eGFR rate of decline (Table 2). These rates of eGFR decline for each imaging class are similar to those reported by Irazabal et al.^9^ A similar association between TKV growth and eGFR decline was demonstrated in the TEMPO 3:4 trial, providing further validation for the model.^19^ This also suggests that a larger relative reduction in eGFR rate of decline may be observed in patients with more rapid TKV progression. For example, in a population of patients progressing at a rate of 5%/year, a 50% reduction in TKV growth rate (ie, from 5% to 2.5%/year) would reduce the eGFR rate of decline from 2.71 to 2.08 mL/min/1.73 m^2^/year (23% reduction; 95% confidence interval [CI], 17%-29%). Furthermore, in patients progressing at a rate of 8%/year, a 50% reduction in TKV growth rate (ie, from 8% to 4%/year) would reduce the eGFR rate of decline from 3.46 to 2.46 mL/min/1.73 m^2^/year (29% reduction; 95% CI, 23%-35%).

**Table 2:** Relationship Between TKV Growth Rate and Predicted eGFR Rate of Decline

TKV Growth Rate, %/y	Predicted eGFR Rate of Decline, mL/min/1.73 m^2^/y (95% CI)	Predicted Effect on eGFR Decline With 50% Reduction on TKV Growth Rate	
		eGFR Rate of Decline, mL/min/1.73 m^2^/y (95% CI)	Relative Reduction in eGFR Decline, % (95% CI)
4	2.46 (2.24-2.67)	1.96 (1.66-2.25)	20% (15%-26%)
5	2.71 (2.52-2.90)	2.08 (1.81-2.35)	23% (17%-29%)
6	2.96 (2.78-3.14)	2.21 (1.96-2.46)	25% (19%-32%)
7	3.21 (3.02-3.40)	2.33 (2.10-2.56)	27% (21%-33%)
8	3.46 (3.25-3.68)	2.46 (2.24-2.67)	29% (23%-35%)
9	3.72 (3.46-3.97)	2.58 (2.38-2.78)	30% (25%-36%)
10	3.97 (3.67-4.26)	2.71 (2.52-2.90)	32% (26%-37%)

*Note*: Predicted eGFR rate of decline for a given TKV growth rate was based on a linear mixed-effect model of eGFR at time t as a function of baseline eGFR, age, and TKV growth rate.

Abbreviations: CI, confidence interval; eGFR, estimated glomerular filtration rate; TKV, total kidney volume.

### STAGED-PKD Study Design

STAGED-PKD is a randomized, double-blind, placebo-controlled, 2-stage study using a seamless design to characterize the efficacy, safety, tolerability, and pharmacokinetics of venglustat in patients at risk of rapidly progressing ADPKD. This efficient trial design combines 2 stages into 1 trial, and data from stage 1 are used in the analysis for stage 2. This is possible because of the similar endpoints in stages 1 and 2 and the identical inclusion/exclusion criteria for the primary analysis population.

#### Stage 1

**Figure 4:**
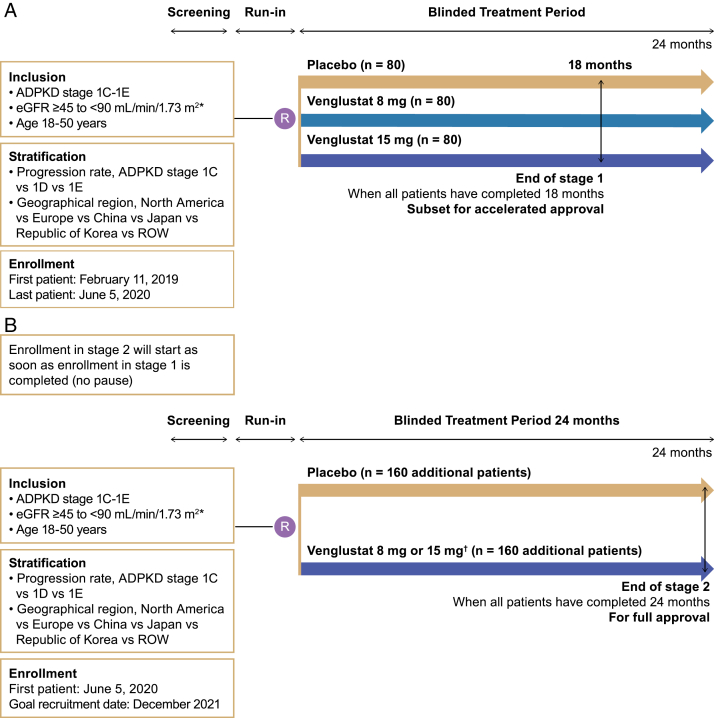
Study design and key steps in STAGED-PKD stage 1 (A) and stage 2 (B). ^∗^To ensure adequate representation of patients across the spectrum of eGFR, a minimum of 20% of patients are enrolled in each of the following categories: ≥45-<60 mL/min/1.73 m^2^; ≥60-<75 mL/min/1.73 m^2^; and ≥75-<90 mL/min/1.73 m^2^; ^†^Highest dose determined to be well tolerated in stage 1. Abbreviations: ADPKD, autosomal dominant polycystic kidney disease; eGFR, estimated glomerular filtration rate; ROW, rest of the world; STAGED-PKD, study to assess glucosylceramide synthase inhibitor efficacy in ADPKD.

The primary objective of stage 1 is to determine the effect of venglustat on the rate of TKV growth in patients at risk of rapidly progressing ADPKD. Because both TKV growth and height-adjusted TKV growth are deemed to be the same in an adult population (and therefore of stable height), height-adjusted TKV was not used. Stage 1 has a ≤30-day screening period, including a 2-week single-blind placebo run-in (to assess compliance in potential patients, ie, identify those unlikely to follow the assigned treatment regimen), followed by a 24-month randomized, double-blind, placebo-controlled treatment period. After run-in, patients are randomized 1:1:1 to receive placebo or venglustat 8 mg or 15 mg once daily for 24 months, with each treatment arm having approximately 80 patients (Fig 4A). The end of stage 1 is defined as completion of 18 months of treatment by all patients (or discontinued), with TKV and eGFR assessed for ≥18 months. Patients from stage 1 will continue blinded treatment for a further 6 months, to a total of 24 months, to obtain eGFR data at the 24-month timepoint.

#### Stage 2

The primary objective of stage 2 is to determine the effect of venglustat on eGFR in patients at risk of rapidly progressing ADPKD. Stage 2 has a similar design to stage 1 (Fig 4B). However, after run-in, approximately 320 additional patients are randomized 1:1 to receive placebo and venglustat (dose determined in stage 1). Thus, with patients from stage 1, each treatment arm will have approximately 240 patients (ie, 80 patients from the stage-1-chosen venglustat dose arm plus 160 patients added at stage 2 versus 240 patients in the placebo arm). Enrollment into stage 2 starts immediately after completion of enrollment into stage 1. After the first 150 randomized patients from stage 1 complete ≥1 month of treatment or prematurely discontinue treatment, the Data Monitoring Committee (DMC) performs an unblinded review of the aggregate safety data to select the venglustat dose for patients in stage 2.

In stage 2, a secondary analysis population of 80 additional patients (aged greater than or equal to 18 to less than or equal to 55 years, baseline eGFR ≥30-<45 mL/min/1.73 m^2^) are randomized 1:1 to venglustat or placebo to assess treatment exposure in patients with more advanced ADPKD. These patients will be analyzed separately from the primary safety and efficacy analyses.

### Endpoints and Assessments

#### Efficacy

The stage 1 primary endpoint is the annualized rate of TKV change based on magnetic resonance imaging from baseline to 18 months (measured at baseline, months 1 [to detect any hemodynamic effect, as was noted for tolvaptan], 9, and 18).^21^ All study sites should use magnetic resonance imaging T2 sequence with fat saturation in the coronal viewing plane. The main stage 1 secondary endpoint is the annualized rate of change in eGFR from baseline to 18 months (measured at baseline, month 1, month 3, and then every 3 months). Creatinine is measured using the Roche Creatinine (rate-blanked) Jaffe method standardized to the isotope-dilution mass spectrometry method.

The stage 2 primary endpoint is the annualized rate of change in eGFR from baseline to 24 months (measured at baseline, month 1, month 3, and every 3 months thereafter). Although measured glomerular filtration rate is the gold standard for measuring kidney function, longitudinal changes in measured glomerular filtration rate and eGFR have been found to be similarly associated with chronic kidney disease-relevant outcomes.^22^ Stage 2 secondary endpoints include the annualized rate of change in TKV based on magnetic resonance imaging from baseline to 18 months. Reversible, negative acute treatment effects on eGFR have been observed in ADPKD clinical trials, and total eGFR slope calculated from prerandomization baselines can be misleading as a result.^17^^,^^23^ The primary analysis in STAGED-PKD assumes no acute effect, and the slope is based on all data from baseline to month 24; a secondary analysis includes only data after 1 month. Our preclinical data and clinical trials in other indications do not support any hemodynamic effect on eGFR. Further, the 1-month evaluation will provide the additional evidence that venglustat has no hemodynamic effect on eGFR. Other secondary endpoints are shown in Table 3. Iohexol is administered in a stage 2 substudy of approximately 15% of patients (eGFR 45-<90 mL/min/1.73 m^2^) to evaluate measured glomerular filtration rate (at baseline, months 12, and month 24). The full schedule of assessments is shown in Table S3. Daily diaries regarding ADPKD symptoms are completed on specified days (Item S4).

**Table 3:** STAGED-PKD Endpoints

Endpoint Type	Endpoint
Stage 1 (from baseline to 18 mo)	
Primary	• Annualized rate of change in TKV based on MRI
Secondary	• Annualized rate of change in eGFR_CKD-EPI_
Stage 2 (from baseline to 24 mo unless otherwise specified)	
Primary	• Annualized rate of change in eGFR_CKD-EPI_
Secondary	• Annualized rate of change in TKV based on MRI from baseline to 18 mo
Stage 1 and stage 2 (from baseline to 18 and 24 mo, respectively)	
Secondary	• Change in pain (BPI-Item 3) • Change in fatigue (BFI-Item 3) • Plasma venglustat concentrations • TEAEs^a^, AEs, and serious AEs • Laboratory parameters, vital signs, electrocardiogram, and physical examination findings • Change in BDI-II score (depression) • Change in lens clarity by ophthalmologic examination^b^, including Snellen or Tumbling E distance chart, slit lamp examination and examination of cornea, lens, and retina

Abbreviations: AEs, adverse events; BDI-II, Beck’s Depression Inventory-II; BFI, Brief Fatigue Inventory; BPI, Brief Pain Inventory; eGFR_CKD-EPI_, estimated glomerular filtration rate chronic kidney disease-epidemiology collaboration; MRI, magnetic resonance imaging; TEAE, treatment-emergent adverse event; TKV, total kidney volume.

a TEAEs are defined as AEs that developed, worsened (in the opinion of the Study Investigator), or became serious during treatment (the period from the first dose to up to 30 days after the last dose of study treatment).

b Examination should include pupil dilation and evaluation of the lens according to the lens opacities classification system III.

#### Safety

Adverse events, vital signs, and laboratory parameters are evaluated at every study visit (Table S3). Full physical examinations are carried out at screening and months 18 and 24, and abbreviated physical examinations (at run-in, baseline, and months 1, 3, 6, 9, 12, 15, 21, and 25) focus on areas important for the assessment of adverse events (Item S5).

Patients who prematurely and permanently discontinue study medication will return for site visits at month 18 (for magnetic resonance imaging and other planned assessments) and month 24 (for eGFR and measured glomerular filtration rate measurement and other planned assessments; Item S6).

### Selection of Study Dose and Study Blinding

Venglustat doses 8 mg and 15 mg were selected for stage 1 assessment based on results from assessments in animal models and healthy volunteers.^5^^,^^24^ Venglustat is provided as 2 × 4 mg or 1 × 15 mg opaque hard gelatin oral capsules; placebo is provided in oral identical capsules in matched packaging (Items S7 and S8). In stage 2, patients will be randomized to the venglustat dose determined by the DMC. If the DMC recommends the 8 mg dose of venglustat for stage 2, patients receiving 15 mg venglustat in stage 1 will switch over to the recommended 8 mg dose for the remainder of the study. However, if the DMC recommends the 15 mg dose of venglustat for stage 2, then patients from stage 1 receiving the 8 mg dose in stage 1 will remain on 8 mg until the end of the study. Irrespective of the dose recommended by the DMC for stage 2, all patients in stage 1 will continue with venglustat treatment for the entire 24 months.

### STAGED-PKD Eligibility Criteria

Patients included in stage 1 and stage 2 are males and females with ADPKD aged greater than or equal to 18 and less than or equal to 50 years with an eGFR ≥45-<90 mL/min/1.73 m^2^ at screening. Eligibility based on eGFR values is confirmed at 1 of the 2 first prerandomization visits. All included patients are required to be of Mayo Imaging Class 1C, 1D, or 1E, with TKV values confirmed by a central reader before visit 3 (baseline).^9^

Overt proteinuria is uncommon in patients with ADPKD and therefore is not included as an eligibility criterion in STAGED-PKD; however, proteinuria is measured in all patients.^25^ All patients are screened for comorbid conditions at study entry (Item S9).

All patients are required to give voluntary written informed consent, and study oversight is conducted by a steering committee and DMC independent of the study sponsor (Item S10).

### Statistics

#### Power and Sample Size Calculation

Sample size calculations for stage 1 require randomization of approximately 240 patients 1:1:1 to placebo, venglustat 8 mg once daily, or venglustat 15 mg once daily (n = 80 per arm). Stage 2 requires approximately 320 additional patients randomized 1:1 to placebo or venglustat preferred dose for the primary analysis. This is sufficient to provide approximately 89% power to detect a 50% reduction in the annualized rate of change in TKV based on stage 1 data and approximately 87% power to detect a 30% reduction in the annualized rate of change in eGFR between venglustat and placebo based on combined stage 1 and stage 2 data. Overall, the total sample size provides approximately 87% power to detect an effect on both TKV and eGFR (Item S11).

#### Statistical Analysis

An interim analysis for futility will be performed when all patients from stage 1 have completed 9 months of treatment and approximately 30% have completed 18 months of treatment with TKV data available (or have prematurely discontinued). Futility may be declared if insufficient effect of venglustat on the annualized rate of change in TKV is observed at this interim analysis, based on prespecified but nonbinding criteria. Futility may be declared if the 1-sided *P* value of the primary endpoint at the interim analysis is >0.30. The 1-sided *P* value will be determined from the Multiple Comparison Procedure. Based on simulations, it is expected that futility may be declared if the relative reduction versus placebo in TKV growth rate estimated at the interim analysis is less than 15%. The interim analysis will focus on the primary endpoint in stage 1 (annualized rate of change in TKV) and stopping rules will be based on this primary endpoint.

Stage 1 data will be analyzed when all patients from stage 1 have been randomized and all data are available up to month 18. Stage 2 analysis will include combined stage 1 and stage 2 data available from baseline to the end of the 24-month treatment period. The primary analysis of stage 1 data will be conducted on all randomized patients in stage 1 (intent-to-treat population). The primary analysis of combined stage 1 and stage 2 data will include all patients with an eGFR ≥45-<90 mL/min/1.73 m^2^ at screening randomized in stage 1 and stage 2 (intent-to-treat population).

For analysis of the primary endpoint in stage 1 (annualized rate of change in TKV), a linear mixed-effect model will be fitted to the log_10_-transformed TKV, including fixed effects of treatment (venglustat 8 mg, venglustat 15 mg, or placebo), time (as a continuous variable), and treatment × time interaction (Item S12). In the primary analysis, TKV slopes will be estimated using all data from baseline to month 18. A secondary analysis will explore any potential acute effect and will exclude data during the first month.

The primary endpoint in stage 2 is the annualized rate of change in eGFR. The analysis of eGFR will be similar to that of TKV, though without log transformation. The multiplicity of endpoints (TKV and eGFR) and multiplicity of analysis (at the end of stage 1 and stage 2) will be handled using a prespecified statistical procedure, ensuring a strong control of the overall Type I error rate at the 0.05 level for the entire study. The statistical procedure is illustrated using a graphical approach and was discussed and agreed on with the Food and Drug Administration.^26^ The protocol received regulatory approval in over 20 countries globally.

## Discussion

STAGED-PKD was developed using prognostic enrichment strategies based on modeling to overcome the complexities of designing an interventional trial in patients with ADPKD. This approach is necessary because of the prolonged time during which kidney function is intact while disease is progressing (ie, cyst burden is increasing). Combining the characteristics of high cyst burden and declining eGFR in a relatively young cohort enables to test the hypothesis that potential GCS inhibition by venglustat may slow cyst growth and preserve kidney function. The seamless 2-stage design of STAGED-PKD is because of the similar endpoints in stage 1 and stage 2 and the identical inclusion/exclusion criteria for the primary analysis population in both stages. Stage 1 uses a nested approach, where the assessment of TKV in stage 1 allows for early efficacy evaluation and improves trial design efficiency by reducing the sample size and decreasing overall duration. However, it also means that, if the study fails the TKV endpoint, the eGFR endpoint cannot be reached.

The statistical modeling described herein suggests that, in a population of patients with rapidly progressing ADPKD, a 50% reduction in TKV growth rate would constitute a substantial improvement and may be associated with an approximate 30% reduction in eGFR rate of decline. A clinical trial with TKV growth rate as a primary endpoint should therefore be appropriately powered to detect a 50% reduction in the TKV growth rate. Statistical modeling also suggests that a larger effect on eGFR may be observed in patients with more rapid progression of TKV growth, justifying the enrichment of the STAGED-PKD trial with patients from Mayo Classes 1C-1E. As a result, STAGED-PKD was designed to use an enrichment strategy to identify blended early- and late-stage patients who are likely to have rapidly progressing ADPKD.^8^ Of note, study powering to achieve an approximate 50% treatment effect on TKV growth rate (actually a 49.2% reduction) was achieved in TEMPO 3:4, resulting in a 30% slowing of the eGFR decline rate, with a subsequent post hoc exploratory analysis indicating that patient enrichment would have increased study power and efficiency.^19^^,^^27^

Several studies have reported a significant effect on TKV without significant effects on eGFR. Specifically, HALT-PKD, ALADIN 1 and 2, and DIPAK 1 reported modest reductions in TKV growth rate ranging from 15%-37%, with little or no amelioration of kidney function decline.^17^^,^^28^, ^29^, ^30^ However, based on the present model, effects on TKV growth of this magnitude would not be expected to translate into major effects on eGFR; therefore, STAGED-PKD targeted a 50% reduction in TKV growth in stage 1. Furthermore, in ALADIN 1, the reduction in TKV growth was found only in the first year after treatment; follow-up scans at 3 years showed no benefit of treatment on TKV growth.^28^ The lack of effect on eGFR in the DIPAK 1 and ALADIN 1 and 2 trials could be due to various reasons, including an intrinsic nephrotoxic effect of the drug offsetting any potential benefit, administration of a dose too low to impact eGFR decline, and inclusion of patients with later-stage ADPKD in whom TKV growth has a smaller impact on eGFR decline compared with patients with earlier-stage ADPKD.^29^ Of note, in a post hoc analysis of the HALT-PKD Blood Pressure trial, where only the chronic eGFR slope was considered (data from 4 months, excluding an acute, reversible eGFR reduction seen as a result of achieving the low blood pressure target), the difference between rigorous and standard blood pressure control reached exactly *P* = 0.05, with a 0.4 mL/min/1.73 m^2^ difference in the rate of kidney function decline and a 14.2% difference (*P* = 0.006) in the rate of kidney volume increase between the 2 treatment groups.^17^

The 2-stage design of STAGED-PKD allows for a time-saving, efficient trial with the possibility of accelarated approval after stage 1 and lowers cost by reducing the overall number of patients needed. Without this study design, STAGED-PKD would have required 600 patients for stage 2 to reach the same statistical power; this would be an additional 200 patients than planned. Despite these advantages, there is a risk associated with the 2-stage design; if the intervention is unsuccessful, as determined by the stage 1 futility analysis, patients may develop venglustat-related adverse events that can have long-lasting effects and possibly require medical interventions, without gaining any drug-related benefit. Other challenges associated with the 2-stage design are development of validated statistical methods and adequate sample size requirements for achieving the desired power for the study objectives.^31^

The pharmacokinetics of venglustat have been assessed in healthy individuals and animal models and described in detail previously.^4^, ^24^

STAGED-PKD tests a novel biological mechanism in the treatment of ADPKD, facilitating a better understanding of the function of GSLs in this disease. The molecular basis of GSL accumulation in ADPKD is not well understood, but the mammalian target of rapamycin pathway activation downstream of polycystin/primary ciliary dysfunction has been implicated, resulting in increased GCS activity and de novo ceramide synthesis.^4^ GSL accumulation may also promote cyst growth by perpetuating aberrant signaling via the primary cilium beyond the effects of ADPKD.^4^ As GCS is the target, or is downstream in the lipid biosynthesis pathway of aberrant signaling in patients with ADPKD, the GCS inhibitor venglustat may have the potential to attenuate GSL production and slow cyst formation (Fig 1).

One potential limitation of this study is the absence of an active control. Tolvaptan was not used, as a range of characteristic associated adverse events, a requirement for frequent transaminase monitoring, and the potential for study dropouts make its utility as a comparator challenging in a randomized, blinded study. Moreover, tolvaptan is not available or reimbursed in all of the countries included in STAGED-PKD, and in the other countries, many of the eligible patients with ADPKD do not opt or are not prescribed tolvaptan.^32^ Given these considerations, not using an active control arm was deemed acceptable according to the Investigational Review Boards of participating sites.

In conclusion, this manuscript describes the rationale and design of STAGED-PKD, a seamless 2-stage trial enabling optimal dose selection and evaluation of venglustat safety and efficacy in patients with ADPKD. Patient enrichment alongside the use of change in the TKV growth rate as a surrogate endpoint in STAGED-PKD should result in a more efficient (shorter and smaller) trial than permitted previously.

## Supplementary Material

Supplementary File 1 (PDF) Fig S1, Item S1-S12, Table S1-S5.
