# Supplementary material for: The STAGED-PKD 2-Stage Adaptive Study With a Patient Enrichment Strategy and Treatment Effect Modeling for Improved Study Design Efficiency in Patients With ADPKD
Source: Kidney Med. 2022 Aug 27;4(10):100538. doi: 10.1016/j.xkme.2022.100538 (PMC9529969; doi:10.1016/j.xkme.2022.100538)
Supplement: Supplementary File 1 (PDF) — Fig S1, Item S1-S12, Table S1-S5. [file mmc1.pdf]

Figure S1. Flowchart of the Study Population Included in the Retrospective Analysis of CRISP and HALT-PKD A Studies

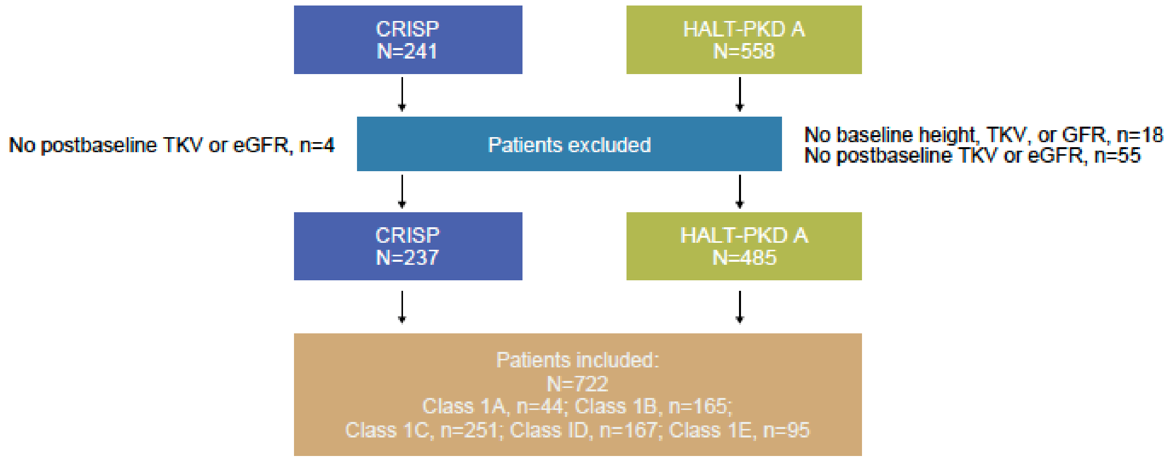

CRISP, Consortium for Radiologic Imaging Studies of Polycystic Kidney Disease; eGFR, estimated glomerular filtration rate; HALT-PKD, HALT Progression of Polycystic Kidney Disease; TKV, total kidney volume

Item S1: Study Population Enrichment

In autosomal dominant polycystic kidney disease (ADPKD), patients at risk of rapid progression have been difficult to identify, as manifestations differ substantially with disease severity. Moreover, despite recent results of positive treatment outcomes in patients at late disease stages,[1] it seems intuitive to select patients with relatively early disease (preserved kidney function and low total kidney volume [TKV]), who may derive most treatment benefits due to a potentially larger impact of TKV growth on estimated glomerular filtration rate (eGFR) decline.[2] However, patients with relatively early disease are usually asymptomatic and may not progress during the study timeframe. Patient enrichment for rapid progressors among a blended early and late-stage population was therefore deemed necessary.

The Mayo ADPKD Imaging Classification system is used to define patients with ADPKD by their risk of eGFR decline, based on height-adjusted TKV (htTKV) at baseline in relation to age.[3] Only individuals with typical ADPKD characterized by cysts throughout the kidneys are considered Class 1; those with asymmetric, lopsided, unilateral, or atrophic disease progress more slowly.[3] The Mayo model assumes a theoretical starting htTKV of 150 mL/m and a constant yearly increase in htTKV from birth to baseline for those with typical ADPKD. Using these assumptions, patients are classified as either: Class 1A, yearly increase rate <1.5%; Class 1B, rate 1.5–3%; Class 1C, rate 3–4.5%; Class 1D, rate 4.5–6%; or Class 1E, rate >6%. Patients in Mayo Classes 1A and 1B are considered at low and intermediate risk of eGFR decline and are not good candidates for trial inclusion (although longitudinal follow-up is warranted). Patients in Mayo Classes 1C to 1E are at high risk of rapid disease progression, may benefit from treatment aimed at reduced cystic growth, and make good candidates for clinical trials.

The Consortium for Radiologic Imaging Studies of Polycystic Kidney Disease (CRISP) study initiated research into the use of TKV as a biomarker in ADPKD. CRISP 1 and CRISP 2 measured cyst and kidney growth over 8 years using magnetic resonance imaging (MRI) in 241 patients with ADPKD with creatinine clearance of ≥70 mL/min (NCT1039987) and demonstrated that higher rates of kidney enlargement were associated with a significantly greater decline in eGFR. This indicated that cyst growth promotes decline in kidney function and therapies that target cyst growth therefore have the potential to preserve kidney function.[4] CRISP 3 and CRISP 4 were not included in the analysis because CRISP 4 is still ongoing. The randomized HALT Progression of Polycystic Kidney Disease (HALT-PKD) initiative evaluated the effect of rigorous vs standard blood pressure control with angiotensin-converting enzyme inhibitors and angiotensin II receptor blockers on the rate of TKV growth and eGFR decline in: 558 patients with early ADPKD, defined as baseline eGFR >60 mL/min/1.73 m^2 (HALT-PKD Study A; NCT00283686), and 486 patients with late ADPKD, defined as eGFR 25–60 mL/min/1.73 m^2 (HALT-PKD Study B; NCT01885559).[5,6]

Item S2: Patient Classification for Modeling the Relationship Between TKV Growth and eGFR Decline

Mayo Imaging Classification of patients as having typical (Class 1) or atypical (Class 2) ADPKD was performed in the published studies using prespecified imaging findings. However, imaging classifications for individual subjects were not available in the databases used in the retrospective analysis and, therefore, atypical patients could not be excluded. As patients of Mayo Imaging Class 2 represent 5.4% of the ADPKD population,[7] it was considered that this limitation would have a negligible impact. Patients were then further subclassified into Mayo Imaging Classes 1A, 1B, 1C, 1D, or 1E based on htTKV and age as recorded in the databases (**Table S1; Fig S1**). This database did not include whether an individual was included in both CRISP or HALT-PKD, resulting in a very low possibility of introducing bias in the data.

Item S3: Model Used for Retrospective Analysis of Individual Data From CRISP and HALT-PKD Study A

For assessment of individual TKV growth rate and individual eGFR rate of decline

Individual TKV growth rates were calculated using a linear mixed-effect model on log-transformed TKV, including the fixed effect of time (as a continuous variable) and adjustment for age and Mayo Class at baseline. The model also included random intercept and slope. Predicted individual slopes were obtained using empirical best linear unbiased predictors and back-transformed to obtain individual TKV growth rate (in % per year). Individual rates of decline in eGFR were calculated similarly (with no logarithmic transformation). The use of linear predictors in the analysis of the eGFR rate of decline was deemed appropriate over the relatively short duration of this clinical trial; nonlinearity would need to be considered if life-time modeling were performed.

Statistical model to predict future eGFR at time t

This model was based on a linear mixed-effect model, including the fixed effect of time since baseline eGFR (in years), baseline eGFR (in mL/min/1.73m²), age at baseline (in years), TKV growth rate (in %/year) as well as the interaction terms of baseline eGFR by time, age at baseline by time, and TKV growth rate by time. The model also included random intercept and slope. The model can be written as (where u_0 and u_1 are individual random intercept and slope; please see **Table S2** for definitions of parameters):

$$eGFR_t = \beta_0 + \beta_1 \times eGFR_{base} + \beta_2 \times Age + \beta_3 \times \text{TKV growth rate} + \beta_4 \times time + \beta_5 \times eGFR_{base} \times time + \beta_6 \times Age \times time + \beta_7 \times \text{TKV growth rate} \times time + u_0 + u_1 \times time$$

Predicted annualized eGFR rates of decline associated with TKV growth rates varying from 4%/year to 10%/year were obtained from the model. The relative reduction in eGFR rate of decline associated with a reduction in TKV growth rate varying from 0% (no reduction in TKV growth rate) to 100% (complete halt of TKV growth) were obtained from this model. Confidence intervals were obtained using a first-order Taylor approximation for estimating the variance of relative reduction.

Item S4: ADPKD Symptom Diary

The ADPKD symptom diary will be completed on specified consecutive days before the baseline visit, from the baseline visit to Month 3, and before the site visits at Month 12, 18, and 24. The diary comprises three items plus an additional item to log pain medication use, namely: (1) a nocturia item, 'Last night, how many times did you wake up because you had to urinate'; (2) Item 3 of the Brief Fatigue Inventory – Short Form, 'Please rate your fatigue by circling one number that best describes your WORST level of fatigue over the last 24 hours' from 0 (no fatigue) to 10 (fatigue as bad as you can imagine); (3) Item 3 of the Brief Pain Inventory – Short Form, 'Please rate your pain by marking the box beside the number that best describes your pain at its worst in the last 24 hours' from 0 (no pain) to 10 (pain as bad as you can imagine); (4) medication log, 'Have you taken any medication for your pain in the past 24 hours; if so, what was this medication?'

Item S5: Safety Endpoints

Venglustat crosses the blood–brain barrier in *in vivo* models of Gaucher disease and Fabry disease, with modification of neuropathic and behavioral manifestations in Gaucher disease.[8,9] In the open-label, single-arm, Phase 2 venglustat study in 11 patients with Fabry disease, treatment-emergent adverse events (TEAEs) in the nervous system disorders and psychiatric disorders system organ classes occurred in 54.5% and 27.3% of patients, respectively, including depressed mood in two patients (18.2%).[10] Patients with ADPKD have a high prevalence of depression with 22–60.5% of patients having symptoms of depression.[11,12] In STAGED-PKD, neurologic and psychiatric adverse events (AEs) are closely monitored via standard neurologic examination and use of the Beck Depression Inventory-II (BDI-II).

Reversible lens degeneration of unknown pathogenesis with venglustat administration in juvenile rats and mild lenticular opacities in one patient in the venglustat Phase 2 Fabry disease study (where lens opacities are part of the disease natural history)[10] resulted in active monitoring via periodic ophthalmologic examinations in STAGED-PKD (and exclusion of patients with certain types of cataracts) (**Tables S3 and S4**).

Item S6: Treatment Discontinuation

If, during the study, a patient experiences a reduction in eGFR to <30 mL/min/1.73 m^2 on two separate visits, they will be discontinued from the study due to the possibility of increased exposure to venglustat (beyond the range in healthy individuals) as a result of impaired kidney function. If eGFR is <25 mL/min/1.73 m^2, a repeated test should be performed within 30 days; if the result is <30 mL/min/1.73 m^2, the patient will be discontinued from the study.

Patients who prematurely and permanently discontinue study medication in Stage 1 or Stage 2 will complete an end-of-treatment assessment visit within 7 days, which should include all Visit 12 (Month 24) assessments. A follow-up visit 30 days later will also be performed, and the patient will continue study visits for scheduled assessments if possible (or continue to complete their ADPKD symptoms diary).

Item S7: Study Blinding Procedure

In Stage 1 during the run-in period, patients receive placebo matching venglustat (2 capsules) once daily (QD) for 2 weeks. During the Stage 1 double-blind treatment period, patients receive venglustat 8 mg QD (2 capsules of venglustat 4 mg each), venglustat 15 mg QD (1 capsule of venglustat 15 mg, 1 placebo capsule), or placebo (2 capsules) for 24 months. In Stage 2 during the run-in period, the patient receives placebo matching venglustat QD for 2 weeks (1 or 2 capsules depending on the dose of venglustat selected for Stage 2 during unblinded review of Stage 1 aggregate safety data by the Data Monitoring Committee [DMC]). During the Stage 2 double-blind treatment period, patients receive placebo or venglustat QD at the selected dose for 24 months.

Patients, Investigators, and study site personnel will remain blinded to study treatment with no access to the randomization list, unless an AE occurs where knowledge of the investigational medicinal product is required for treating the patient or is required by the local regulatory authorities. If emergency unblinding occurs at the site level by the Investigator, then the patient is withdrawn from treatment. If the unblinding occurs at the study level by the Sponsor, then the patient is not withdrawn from treatment.

Item S8: Diet and Other Considerations

Based on results of a Phase 1 study in healthy volunteers, which indicated that a high-fat meal had no effect on venglustat exposure,[13] venglustat or placebo is taken around the same time each day throughout the study and can be administered without restriction to food other than avoiding grapefruit-containing products within 72 hours of starting venglustat treatment and throughout the remainder of the study.

In general, it is advised to reduce dietary sodium intake to within limits recommended for the general healthy population and general chronic kidney disease (CKD) population (<100 mmol/d). Increased fluid intake is not recommended, since urinary osmolality is an endpoint in the STAGED-PKD trial.

Patients are required to take their study medication onsite at visits where a postdose pharmacokinetics sample is to be collected. Treatment compliance is monitored by the return of assigned treatment kits (empty or unused) at each visit and by patient compliance diary. Venglustat interruptions are permitted for patients who require the use of strong or moderate CYP3A4 inhibitors or inducers for ≤2 weeks on no more than two occasions.

Item S9: Eligibility Criteria

Diagnosis of ADPKD in patients with a family history are based on modified Pei criteria;[14] in patients without a family history, the diagnosis is based on the presence of ≥20 bilateral kidney cysts in the absence of evidence suggestive of other cystic kidney diseases. All potentially eligible patients who are hypertensive are required to be on stable antihypertensive treatment for ≥30 days before the screening visit. At the start of the study, patients must not have access to tolvaptan nor be eligible for tolvaptan treatment according to their physician, based on recommended criteria, refusal to initiate, or inability to tolerate tolvaptan.

There have been no studies of venglustat in pregnant women, and it is not known whether venglustat is secreted in human milk. Therefore, pregnant or lactating women are excluded from STAGED-PKD. Female patients of childbearing potential must have a negative blood pregnancy test at the screening visit and a negative urine pregnancy test at baseline. Males and females of childbearing potential must practice abstinence or use double-contraceptive methods for the whole duration of the study and for ≥6 weeks for females and ≥90 days for males following their last dose of venglustat. Any woman who becomes pregnant during the study will discontinue treatment and continue to be followed.

Patients are excluded if they have systolic blood pressure >160 mmHg at both the run-in visits and baseline. Patients are also ineligible if they have received tolvaptan or somatostatin analogs within 3 months of screening or are participating in another investigational interventional study or using an investigational medicinal product within 3 months or 5 half-lives (whichever is longer) before randomization. Participation in STAGED-PKD is not permitted if the patient cannot adhere to study requirements or undergo the necessary study assessments, such as MRI.

Patients are excluded if they are unable to adhere to treatment (demonstrate a >70% compliance rate during run-in).

Additional exclusion criteria based on clinical parameters, concomitant medication use, and risks identified during the nonclinical toxicology studies are shown in **Table S4**.

Item S10: Ethics and the Role of the Steering Committee and Data Monitoring Committee

All patients are required to give voluntary written informed consent before performance of any study-related procedures, and all patients must be able to read, comprehend, and respond to study questionnaires. The study is conducted in accordance with consensus ethics principles derived from international ethics guidelines, including the Declaration of Helsinki and the International Council for Harmonisation guidelines for good clinical practice. The clinical trial protocol was submitted to the health authorities and appropriate institutional review board/independent ethics committee for each study center (IRB study code: Pro00028299).

The Steering Committee comprises field experts and Sponsor-based scientists with clinical and methodological expertise. This committee will: provide advice to the Sponsor regarding scientific issues and operational conduct of the study; review any protocol amendments; receive blinded study status reports from the Sponsor; review DMC recommendations throughout the study; and provide input regarding the interpretation of study results. The members will remain blinded until completion of the study.

A DMC independent of the study Sponsor and Investigators will oversee patient safety and assessment of risk:benefit throughout the study. The DMC comprises external experts in ADPKD, biostatistics, and/or clinical research and will be responsible for selecting the single venglustat dose for Stage 2 via review of unblinded safety data from Stage 1. The DMC will supervise an interim analysis for futility that will be performed by the external independent statistician. The DMC will also review the secondary efficacy endpoint (annualized rate of change in eGFR) and safety data (AEs, laboratory data, vital signs) available at the time of the interim analysis.

Item S11: Sample Size Assumptions

Sample sizes are based on a number of assumptions, taken from the retrospective analysis of the CRISP and the HALT-PKD Study A data as part of the modeling used to design STAGED-PKD. Assumptions are an average TKV growth rate of 6.6%/year (slope of $\log_{10}$TKV, 0.02764; standard deviation for the residual error of TKV [on the $\log_{10}$ scale], 0.02566; standard deviation for the random effect of slope, 0.01477) and eGFR rate of decline of –3.66 mL/min/1.73 m^2/year (standard deviation for the residual error of eGFR, 6.34; standard deviation for the random effect of slope, 1.98) in the placebo arm. Sample size and power calculations also assume an overall significance level of 0.05 (2-sided), 10% dropout rate, and include adjustments for the handling of the multiplicity of tests and futility analysis. An additional sample size of 80 patients in Stage 2 with an eGFR between 30 and 44.9 mL/min/1.73 m^2 at screening (not part of the primary analysis) will be required to provide approximately 80% probability to detect a treatment effect in this subgroup at the 0.20 significance level (two-sided), based on a model evaluating potential interaction between treatment and baseline eGFR.

Item S12: Statistical Analysis

Stage 1 Primary Endpoint Analysis

For analysis of the primary endpoint in Stage 1 (annualized rate of change in TKV), a linear mixed-effect model will be fitted to the $\log_{10}$-transformed TKV, including fixed effects of treatment (venglustat 8 mg, venglustat 15 mg, or placebo), time (as a continuous variable), and treatment*time interaction. To account for possible differences ixn TKV between groups, as a major determinant of the rate of disease progression, the model will adjust for Mayo Class (1C, 1D, or 1E) and Mayo Class*time interaction. The model will also include random intercept and slope. Within-group mean slope of $\log_{10}$-transformed TKV will be obtained from the linear mixed effect model. A back-transformation will be applied to obtain the annualized rate of change in TKV (in % per year) within each treatment arm, along with their 95% confidence intervals:

$$\text{Annualized rate of change (\% per year) in TKV in group } j = 10^{\beta_j} - 1$$

where β_j is the mean slope of $\log_{10}$-transformed TKV in group j

The overall effect of venglustat 8 mg or 15 mg will be assessed using a Multiple Comparison Procedure.[15] Multiple trend tests will be performed using optimal contrasts determined from a set of three prespecified candidate models for the dose-response relationship (**Table S5**). Optimal contrasts will apply to the treatment * time interaction term in the linear mixed effect model. *P* values will be adjusted for the multiplicity of doses.

Table S1. Baseline Characteristics in CRISP and HALT-PKD A Studies, by Mayo Imaging Class

	Mayo Class				
	**1A (N=44)**	**1B (N=165)**	**1C (N=251)**	**1D (N=167)**	**1E (N=95)**
Age, years	39.2±8.4	38.8±7.8	36.1±8.3	34.6±8.2	27.6±6.9
Male sex, n (%)	12 (27.3)	58 (35.2)	130 (51.8)	87 (52.1)	56 (59.0)
eGFR mL/min/1.73 m²	96.2±12.9	91.4±15.4	91.8±19.7	88.3±20.7	93.4±23.5
TKV, mL	384±63	645±151	1028±343	1626±639	2084±902
htTKV, mL/m	229±36	380±89	592±193	927±350	1170±502

Values are expressed as mean ± SD.

CRISP, Consortium for Radiologic Imaging Studies of Polycystic Kidney Disease; eGFR, estimated glomerular filtration rate; HALT-PKD, HALT Progression of Polycystic Kidney Disease; htTKV, height-adjusted total kidney volume; SD, standard deviation; TKV, total kidney volume

Table S2. Parameter Estimates of the Model Predicting eGFR at Time t

Parameter	Variable	Estimate (SE)	*P* value
β_0	Intercept	28.48 (3.49)	<0.001
β_1	eGFR at baseline, mL/min/1.73m²	0.8292 (0.0217)	<0.001
β_2	Age at baseline, year	–0.3402 (0.0490)	<0.001
β_3	TKV growth rate, %/year	–0.0966 (0.1137)	0.3954
β_4	Time since baseline, year	–3.033 (0.918)	0.0010
β_5	eGFR at baseline * Time, interaction	0.0145 (0.00570)	0.0111
β_6	Age at baseline * Time, interaction	0.00723 (0.01297)	0.5769
β_7	TKV growth rate * Time, interaction	–0.2514 (0.0296)	<0.001

eGFR, estimated glomerular filtration rate; SE, standard error; TKV, total kidney volume

Table S3. Schedule of Assessments*

	**Screening**	**Run-in**	**Blinded Treatment Period**										**Follow-up (off-treatment)**
**Visit**	**1**	**2**	**3 (Baseline)**	**4**	**5**	**6**	**7**	**8**	**9**	**10**	**11**	**12 (end of treatment)**	**13**
**Month**	**–1**	**–0.5**	**0**	**1**	**3**	**6**	**9**	**12**	**15**	**18**	**21**	**24**	**25**
TKV MRI	1,2			1			1			1,2			
eGFR$_{CKD–EPI}$	1,2	1,2	1,2	1,2	1,2	1,2	1,2	1,2	1,2	1,2	1,2	1,2	
mGFR			2					2				2	
AEs	1,2	1,2	1,2	1,2	1,2	1,2	1,2	1,2	1,2	1,2	1,2	1,2	1,2
Vital signs	1,2	1,2	1,2	1,2	1,2	1,2	1,2	1,2	1,2	1,2	1,2	1,2	1,2
Physical exam	1,2	1,2	1,2	1,2	1,2	1,2	1,2	1,2	1,2	1,2	1,2	1,2	1,2
12-lead ECG		1,2				1,2						1,2	
Laboratory evaluation	1,2	1,2	1,2	1,2	1,2	1,2	1,2	1,2	1,2	1,2	1,2	1,2	1,2
Ophthalmologic assessment		1,2			1,2	1,2	1,2	1,2	1,2	1,2	1,2	1,2	
BDI-II		1,2			1,2	1,2	1,2	1,2	1,2	1,2	1,2	1,2	
GSL markers	1,2			1,2		1,2		1,2		1,2		1,2	1,2
Urinary markers (spot)			1,2	1,2	1,2	1,2	1,2	1,2	1,2	1,2	1,2	1,2	1,2
Serum/plasma biomarkers	1,2			1,2		1,2		1,2		1,2		1,2	1,2
Urinalysis with microscopy	1,2			1,2	1,2	1,2	1,2	1,2	1,2	1,2	1,2	1,2	

Blood sample for plasma venglustat concentration			1[†]	1[‡],2[‡]		1[§]				1[§]		2[§]	
PRO assessments													
Complete daily symptom diary		1,2 (7 days before baseline visit)	1,2 (daily)					1,2 (7 days before visit)		1,2 (14 days before visit)		1,2 (14 days before visit)	
BPI			1,2	1,2	1,2	1,2	1,2	1,2	1,2	1,2	1,2	1,2	
BFI			1,2	1,2	1,2	1,2	1,2	1,2	1,2	1,2	1,2	1,2	
PGIS		1,2	1,2		1,2			1,2		1,2		1,2	
PGIC					1,2			1,2		1,2		1,2	
EQ-5D-5L			1,2		1,2	1,2		1,2		1,2		1,2	

*1 indicates assessments made during Stage 1 of the study; 2 indicates assessments made during Stage 2 of the study. [†]Day 1, 3±1 hour post dose. [‡]Predose and 3±1 hour post dose. [§]Predose sample.

AE, adverse event; BDI-II, Beck's Depression Inventory-II; BPI, Brief Pain Inventory; BFI, Brief Fatigue Inventory; ECG, electrocardiogram; $eGFR_{CKD–EPI}$, estimated glomerular filtration rate chronic kidney disease–epidemiology collaboration; EQ-5D-5L, EuroQol 5 dimensions 5 levels; GSL, glycosphingolipid; mGFR, measured glomerular filtration rate; MRI, magnetic resonance imaging; PGIC, Patient Global Impression of Change; PGIS, Patient Global Impression of Severity; PRO, patient reported outcome; TKV, total kidney volume

Table S4. Exclusion Criteria Based on Clinical Characteristics and Concomitant Medication Use

Category	Exclusion Criteria
Compliance	• Compliance rate ≤70% during run-in
Viral infection	• Documented positive test result for:<br>o Hepatitis B surface antigen*<br>o Antihepatitis C virus antibodies<br>o Anti-HIV 1 and 2 antibodies
Other therapies	• Use of potentially cataractogenic medications, such as corticosteroids, psoralens, typical antipsychotics, or amiodarone, more frequently than EOW<br>• Use of alpha-adrenergic receptor agonist glaucoma medications that may worsen cataracts<br>• Receipt of strong or moderate CYP3A4 inducers or inhibitors within 14 days or 5 half-lives (whichever is longer) of randomization<br>• Use of grapefruit-containing products within 72 hours of starting venglustat administration<br>• Use of investigational medication in any other clinical study<br>• Use of tolvaptan
Existing medical conditions that would put the patient at risk, affect analysis of efficacy and safety, or interfere with study compliance	• Cortical cataracts >one-quarter of the lens circumference[†]<br>• Posterior subcapsular cataracts >2 mm[†]<br>• Scheduled for inpatient hospitalization, including elective surgery, during the study<br>• Uncontrolled diabetes mellitus<br>• Tuberculosis<br>• Uncontrolled thyroid disorder<br>• Clinically significant cardiac arrhythmia<br>• Cushing's disease<br>• Addison's disease
Relating to current knowledge of venglustat	• ALT/AST or total bilirubin >2x ULN unless the patient is diagnosed with Gilbert syndrome[‡]<br>• Presence of severe depression (BDI-II >28) and/or a history of major affective disorder within 1 year of screening<br>• Known hypersensitivity to venglustat or its excipients

*Patients with a positive hepatitis B surface antibody test will be eligible provided they have a negative test for hepatitis B surface antigen and hepatitis B core antibody; patients immune due to natural infection (positive hepatitis B surface antibody, negative

hepatitis B surface antigen, and positive hepatitis B core antibody) will be eligible if they are negative for hepatitis B viral DNA. [†]According to World Health Organization Grading; patients with nuclear cataracts remain eligible for inclusion. [‡]Patients with Gilbert syndrome should have no additional signs or symptoms suggestive of hepatobiliary disease and serum total bilirubin level ≤3 mg/dL with conjugated bilirubin <20% of the total bilirubin fraction.

ALT, alanine aminotransferase; AST, aspartate aminotransferase; BDI-II, Beck's Depression Inventory-II; CKD, chronic kidney disease; EOW, every other week; HIV, human immunodeficiency virus; ULN, upper limit of normal

Table S5. Optimal Contrasts for the Three Prespecified Candidates of Dose-Response Models

Dose	Emax model	Linear model	Sigmoid Emax model
Placebo	-0.8131	-0.7223	-0.6615
Venglustat 8 mg	0.3424	0.0314	-0.0838
Venglustat 15 mg	0.4707	0.6909	0.7453

References

1. Torres VE, Chapman AB, Devuyst O, et al. Tolvaptan in later-stage autosomal dominant polycystic kidney disease. N Engl J Med. 2017;377(20):1930-1942.
2. Meijer E, Visser FW, van Aerts RMM, et al. Effect of lanreotide on kidney function in patients with autosomal dominant polycystic kidney disease: the DIPAK 1 randomized clinical trial. JAMA. 2018;320(19):2010-2019.
3. Irazabal MV, Rangel LJ, Bergstralh EJ, et al. Imaging classification of autosomal dominant polycystic kidney disease: a simple model for selecting patients for clinical trials. J Am Soc Nephrol. 2015;26(1):160-172.
4. Chapman AB, Bost JE, Torres VE, et al. Kidney volume and functional outcomes in autosomal dominant polycystic kidney disease. Clin J Am Soc Nephrol. 2012;7(3):479-486.
5. Schrier RW, Abebe KZ, Perrone RD, et al. Blood pressure in early autosomal dominant polycystic kidney disease. N Engl J Med. 2014;371(24):2255-2266.
6. Torres VE, Abebe KZ, Chapman AB, et al. Angiotensin blockade in late autosomal dominant polycystic kidney disease. N Engl J Med. 2014;371(24):2267-2276.
7. Irazabal MV, Abebe KZ, Bae KT, et al. Prognostic enrichment design in clinical trials for autosomal dominant polycystic kidney disease: the HALT-PKD clinical trial. Nephrol Dial Transplant. 2017;32(11):1857-1865.
8. Ashe KM, Budman E, Bangari DS, et al. Efficacy of enzyme and substrate reduction therapy with a novel antagonist of glucosylceramide synthase for Fabry disease. Mol Med. 2015;21(1):389-399.
9. Marshall J, Sun Y, Bangari DS, et al. CNS-accessible inhibitor of glucosylceramide synthase for substrate reduction therapy of neuronopathic gaucher disease. Mol Ther. 2016;24(6):1019-1029.
10. Deegan P, Germain DP, Goker-Alpan O. Three-year open label phase 2a investigation of venglustat safety and exploratory efficacy in classic Fabry patients. Poster presented at: Annual Symposium of the Society for the Study of Inborn Errors of Metabolism; September 3-6, 2019; Rotterdam, The Netherlands.
11. Simms RJ, Thong KM, Dworschak GC, et al. Increased psychosocial risk, depression and reduced quality of life living with autosomal dominant polycystic kidney disease. Nephrol Dial Transplant. 2016;31(7):1130-1140.
12. de Barros BP, Nishiura JL, Heilberg IP, et al. Anxiety, depression, and quality of life in patients with familial glomerulonephritis or autosomal dominant polycystic kidney disease. J Bras Nefrol. 2011;33(2):120-128.
13. Sanofi-Genzyme. Data on File.2020.
14. Pei Y, Obaji J, Dupuis A, et al. Unified criteria for ultrasonographic diagnosis of ADPKD. J Am Soc Nephrol. 2009;20(1):205-212.

15. Bretz F, Pinheiro JC, Branson M. Combining multiple comparisons and modeling techniques in dose-response studies. Biometrics. 2005;61(3):738-748.
